# Bridging Acellular Dermal Matrix in Abdominal Wall Repair following Radical Resection of Recurrent Endometrioma

**DOI:** 10.1097/GOX.0000000000002603

**Published:** 2020-01-17

**Authors:** Paige N. Hackenberger, Stephen J. Poteet, Jeffrey E. Janis

**Affiliations:** From the * The Ohio State University College of Medicine, Columbus, Ohio; † Department of Plastic Surgery, The Ohio State University Wexner Medical Center, Columbus, Ohio.

## Abstract

The patient is a 31-year-old woman with a history of prior resection of a presumed keloid scar around her Pfannenstiel incision found to be endometrial tissue on final pathology. She presented 5 years later with recurrence of pain and a mass associated with menses despite maximal medical therapy for endometriosis. Computed tomography of her abdomen and pelvis demonstrated an infiltrative soft tissue mass measuring 8.8 cm × 4.0 cm. Surgical oncology conducted an en bloc resection of the mass and obstetrics and gynecology performed a concomitant total abdominal hysterectomy and bilateral salpingo-oophorectomy. Plastic and reconstructive surgery completed the repair of the final 23 cm × 10 cm full-thickness abdominal wall defect with bridging biologic mesh, complex layered closure, and incisional negative-pressure wound therapy. Final pathology confirmed a diagnosis of endometriosis. Patient’s hospital course was uncomplicated, and follow-up at 6 months does not demonstrate clinical or radiographic evidence of bulge or hernia recurrence. Abdominal wall endometrioma is a well-documented occurrence in prior cesarean scars; plastic surgeons can contribute to a multidisciplinary approach in reconstruction when resection compromises abdominal wall integrity, necessitating expertise in complex repairs.

The diagnosis of abdominal wall endometriosis (AWE) is made when endometrial tissue superficial to the peritoneum is confirmed by histopathology. The incidence of cesarean scar endometriosis is estimated between 0.03% and 0.45%, and patients most commonly present with a mass (96%) and pain (87%; 57% of whom experience cyclical discomfort associated with menstruation).^1–3^ The infiltrative nature of endometrial tissue can result in large or recurrent lesions which require wide excision and removal of the bilateral ovaries to achieve an optimal therapeutic outcome. Due to association with the abdomen and prior incision, the differential diagnosis includes incisional hernia, keloid scarring, desmoid tumor, suture granuloma, fat necrosis, or malignancy.

The treatment of choice for AWE is wide local excision with at least 1 cm margins, and recurrence is estimated at 4.3%.^4^ Although AWE is well documented in the literature as a late complication following cesarean section, the average reported size (2.7 cm) typically lends toward primary fascial closure following resection.^3^ In cases of large abdominal wall defects, options for closure include unilateral or bilateral component separation, bridging interposition mesh, or musculofasciocutaneous/fasciocutaneous free flap.

## CASE REPORT

**Fig. 1.**
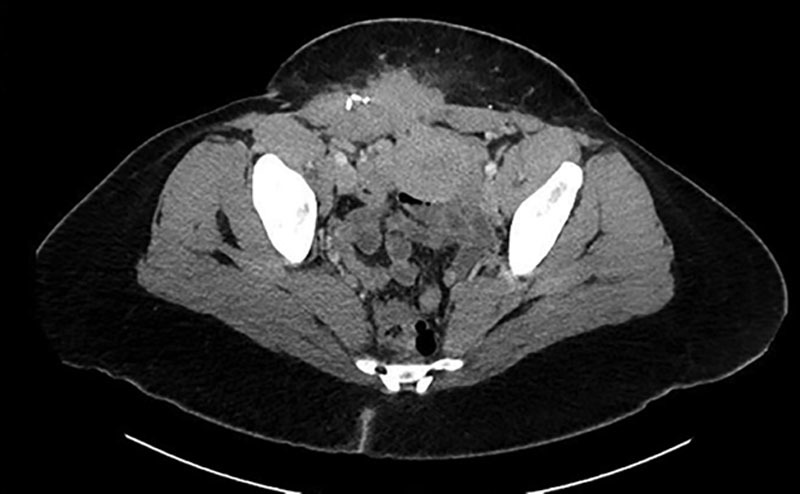
Computed tomography image showing infiltrating mass within abdominal wall.

A 31-year-old African American woman with a history of 2 low transverse cesarean sections and prior resection of abdominal scar endometrioma 5 years ago presented with a recurrent Pfannenstiel incision-associated mass and chronic, severe pain uncontrolled by maximal medical therapy. Computed tomography with contrast of the abdomen and pelvis demonstrated evidence of an infiltrative soft tissue mass involving the right lower quadrant ventral abdominal wall musculature and subcutaneous tissues measuring 8.8 cm × 4.0 cm (Fig. 1). The patient was evaluated by a multidisciplinary team including surgical oncology sarcoma specialists, obstetrics and gynecology, and plastic and reconstructive surgery for surgical planning of wide excision and repair.

**Fig. 2.**
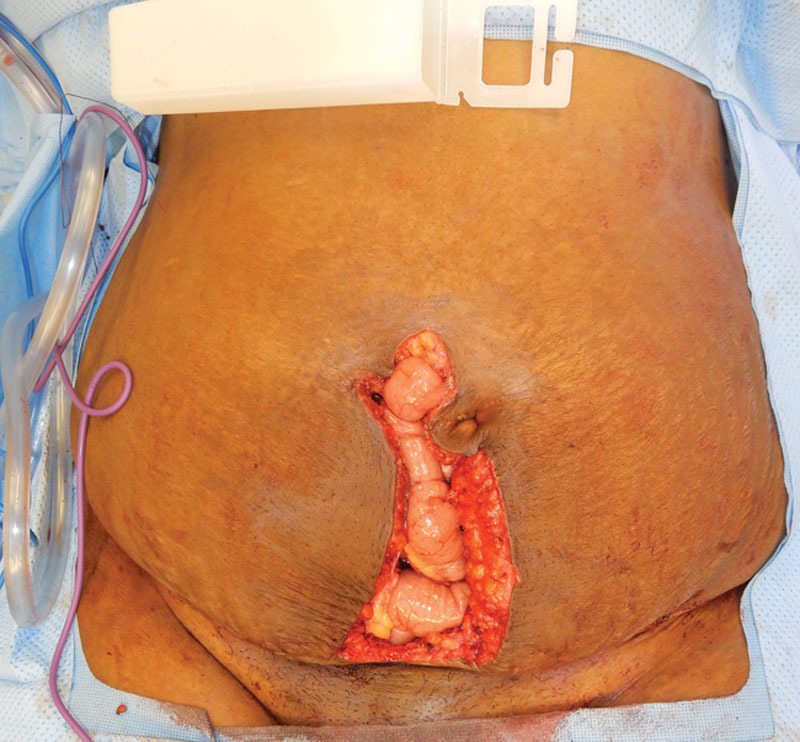
Defect after oncologic resection demonstrating exposed bowel without loss of overlying skin.

**Fig. 3.**
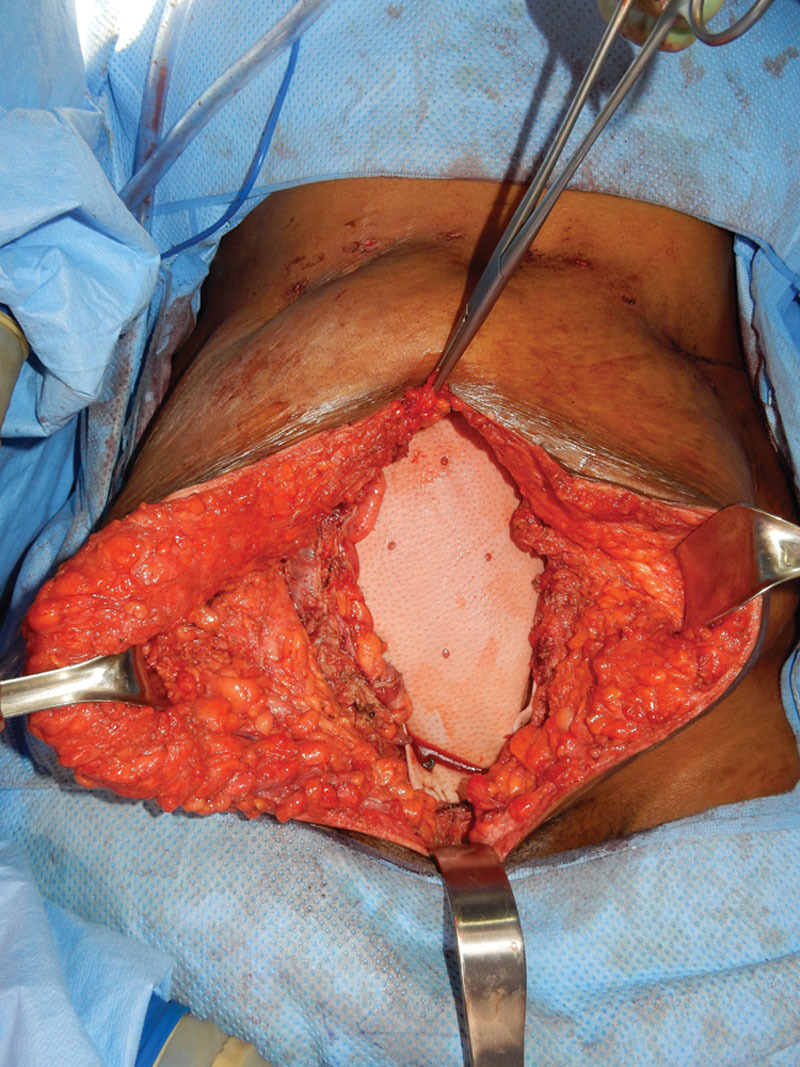
Placement of bridging perforated porcine ADM.

The full-thickness composite musculofascial abdominal wall defect measured 23 cm × 10 cm (Fig. 2) with associated bilateral pubic bone exposure after en bloc resection of the endometrioma and concurrent hysterectomy and bilateral salpingo-oophorectomy. Due to the inherent field contamination associated with the concomitant gynecologic procedure and the proximity to bowel, a 20 cm × 20 cm perforated porcine noncrosslinked acellular dermal matrix (ADM) was selected for the repair and placed as a wide intraperitoneal underlay (Fig. 3). The ADM was secured inferiorly with 5 bone anchors and running long-acting resorbable suture. The most superior 8 cm of the resected musculofascia was primarily reapproximated over the ADM using long-acting resorbable sutures in interrupted, figure-of-8 fashion. The final size of the bridged repair was 15 cm × 8 cm. The skin was closed through design of suprafascial bilateral soft tissue advancement flaps with closure in both interrupted and running fashion. The patient received a kenalog 40 μg/mL injection along the incisional dermal margin due to known history of keloid scarring. Finally, the dermis was reapproximated over a 19-French Blake drain and the closed wound was covered with incisional negative-pressure wound therapy set to 125 mm Hg per surgeons’ standard practice to decrease perioperative complications related to wound healing.

The patient had an uncomplicated postoperative hospital stay and was discharged on postoperative day 5. Follow-up out to 6 months did not demonstrate clinical nor radiographic evidence of hernia recurrence or bulge.

## DISCUSSION

This case presents an interesting challenge in surgical planning and reconstruction due to a multitude of factors: the unknown extent of resection necessary before time of repair, wide inferior defect with pubic bone exposure, inherent contamination associated with concomitant gynecologic procedure, and need for mass pathology and sufficient margin confirmation. This defect, which spans several quadrants of the MD Anderson oncologic abdominal wall reconstruction classifications (type V), typically relies on a bridging mesh repair.^5^ Additionally, due to the inherent field contamination, a biologic ADM was selected to mitigate infection risk.^6^ Finally, the need to confirm mass pathology and sufficient margins on permanent section indicated a possibility of reresection which would risk damage to any permanent mesh and primary fascial closure. The combination of these factors led to the decision to forgo component separation to preserve the patient’s musculofascial integrity for a final repair during which field contamination and pathology could be controlled.

This repair balanced patient-specific factors with plastic surgical principles to minimize morbidity. However, patients receiving abdominal wall reconstruction with use of ADM in the setting of oncologic extirpation have a high likelihood of hernia recurrence (odds ratio = 6.47).^7^ Additionally, bridging mesh predisposes patients to risk of hernia recurrence (56% with biologic mesh used as an interposition bridge) and infection (25%).^8,9^ Therefore, a multidisciplinary approach, adequate patient counseling of expectations, and intraoperative flexibility are needed to address unique complications or scenarios following wide, deep excisions that significantly alter the structural integrity of the abdomen.^10^ Plastic and reconstructive surgeons well versed in complex abdominal wall repairs play an integral role in the treatment of these patients as they can best address current defects and hedge against future complications.

## CONCLUSIONS

Resection and repair of infiltrative AWE require a multidisciplinary approach and intraoperative problem solving by plastic surgeons familiar with complex abdominal wall repairs. Additionally, likelihood of recurrence requires surgical foresight in case of future necessary repairs following additional resections.
